# Increased Pain Symptomatology Among Females vs. Males With Fragile X-Associated Tremor/Ataxia Syndrome

**DOI:** 10.3389/fpsyt.2021.762915

**Published:** 2022-01-20

**Authors:** Devon Johnson, Ellery Santos, Kyoungmi Kim, Matthew D. Ponzini, Yingratana A. McLennan, Andrea Schneider, Flora Tassone, Randi J. Hagerman

**Affiliations:** ^1^Medical Investigation of Neurodevelopmental Disorders Institute, School of Medicine, University of California, Davis, Davis, CA, United States; ^2^Division of Biostatistics, School of Medicine, University of California, Davis, Davis, CA, United States; ^3^Department of Pediatrics, School of Medicine, University of California, Davis, Davis, CA, United States; ^4^Department of Biochemistry and Molecular Medicine, School of Medicine, University of California, Davis, Davis, CA, United States

**Keywords:** FXTAS, pain, premutation, FMRP, fibromyalgia, neuropathy, migraine, anxiety

## Abstract

Individuals with the fragile X premutation report symptoms of chronic pain from multiple systems, have increased incidence of comorbid conditions where pain is a prominent feature, and pathophysiology that supports disrupted pain regulation, inflammation, and energy imbalance. Less is known about how pain manifests for the subpopulation of carriers that develop the motor and cognitive changes of fragile X-associated tremor and ataxia syndrome (FXTAS), and how pain may differ between men and women. We gathered data collected from 104 males and females with FXTAS related to chronic pain, comorbid conditions related to pain, and medications used for pain control to further explore the types of pain experienced and to better characterize how individuals with the fragile X premutation experience pain sensation across genders. We found that women experience significantly more pain symptoms than men, particularly allodynia (20 vs. 2.0%, *p* = 0.008), peripheral neuropathy pain (43.9 vs. 25.4%, *p* = 0.0488), migraine (43.9 vs. 14.5%, *p* = 0.0008), fibromyalgia (26.8 vs. 0%, *p* = 0.0071) and back pain (48.5 vs. 23.4%, *p* = 0.008). We found onset of peripheral neuropathy predicts the onset of ataxia (β = 0.63 ± 0.25, *p* = 0.019) and tremor (β = 0.56 ± 0.17, *p* = 0.004) across gender. Women also report significantly more anxiety (82.9 vs. 39.7%, *p* < 0.001), which has implications for ideal pain treatment. These pain symptoms need to be recognized in the medical history and treated appropriately, with consideration for overlapping comorbidities.

## Introduction

The fragile X premutation is a genetic condition characterized by an increased number of trinucleotide repeats (55–200 CGG repeats) in the *FMR1* gene, which encodes for the fragile X mental retardation protein (FMRP) central for neuronal development and synaptic plasticity in the central and peripheral nervous systems. Expansion of over 200 CGG repeats leads to hypermethylation and gene silencing resulting in the fragile X full mutation and fragile X syndrome (FXS). The premutation leads to enhanced levels of *FMR1* mRNA from 2 to 8 times normal and the excess mRNA causes RNA toxicity including the sequestration of proteins important for neuronal function, oxidative stress, and mitochondrial dysfunction that eventually can lead to the fragile X-associated tremor ataxia syndrome (FXTAS) (1).

FXTAS is a late-onset neurodegenerative condition that occurs in approximately 40% of male and 16% of female carriers. FXTAS affects cognition and movement in individuals, typically males > females over age 50, with core features including: gait ataxia, intention tremor, parkinsonism, executive function deficits, neuropathy and dysautonomia (2). Females with FXTAS typically have less severe movement symptoms (3) and cognitive decline (4), likely because their second X chromosome with normal *FMR1* expression protects the CNS. The percentage of cells with normal allele present on the active X chromosome is the activation ratio (AR) and higher AR may be protective against some manifestations of FXTAS (5). However, females with FXTAS have higher rates of autoimmune conditions and psychiatric manifestations (6, 7). Radiological criteria for the diagnosis of FXTAS include the major findings of white matter lesions in the middle cerebellar peduncles (MCP sign) or brainstem, and the following minor signs: white matter disease in splenium of corpus callosum and/or cerebrum, and moderate-severe brain atrophy (2). Among females with FXTAS there exists high radiological variability; fewer females have the MCP sign (9% in females vs. 60% in males) but have more diffuse cerebral white matter changes, including at the pons (4).

Of significant interest are the high frequency of pain complaints, as well as comorbid conditions with chronic pain that often start prior to FXTAS onset and vary by gender. Peripheral neuropathy is common in FXTAS (3) and can be a presenting feature of the syndrome (8). Musculoskeletal pain is common; back pain (9) and general muscle pain can often lead to the diagnosis of fibromyalgia (10), especially in women where the prevalence of fibromyalgia has been found as high as 43% (11). In the general population, fibromyalgia typically ranges between 2.4 and 6.8% among women (12).

The prevalence of migraine in premutation females has been found to be 54.2 vs. 26.8% in males (13), whereas the prevalence among females in US general population is reported to be 20.7 vs. 9.7% in males (14). In addition, there exists a high prevalence of coexisting conditions related to pain such as psychiatric problems that present in childhood or adulthood (7, 15, 16), sleep problems in up to a third of carriers (17, 18), and executive function deficits (19). These conditions often present prior to the diagnosis of FXTAS and are common.

Pain is generally classified by pain type- nociceptive and neuropathic pain, as well as at different levels-central, spinal cord gating, peripherally (20). Both nociceptive and neuropathic chronic pain are prevalent in individuals with FXTAS. Nociceptive pain is caused by mechanical or chemical damage to a body tissue such as skin, muscles, joints, or fascia; nociceptors signal the brain of the injury, leading to pain perception. Examples of nociceptive pain conditions include arthritis, musculoskeletal disorders, migraine, tension headache, and fibromyalgia. Neuropathic pain can be caused by direct damage to peripheral nerves such as in diabetic neuropathy or at the level of the CNS such as in a stroke, tumor, or neurodegenerative disease like Parkinson's disease or Multiple Sclerosis, which damage areas related to pain. Central sensitization describes a type of neuropathic pain that occurs when pain signals to the brain are pathologically amplified, resulting in a high level of pain stimuli experienced in response to normally non-painful touch stimuli (allodynia) as well as increased feeling of pain stimuli (hyperalgesia). In response, the threshold for sending a signal to the CNS increases resulting in limited signal (21). To compensate for diminished peripheral signal, the spinal cord greatly amplifies the signal it does receive *via* increasing synaptic efficacy of somatosensory neurons in the dorsal horn (22).

Previous research suggests that FMRP has a role in the development of nociceptive pain sensitization and chronic pain (23, 24), and is studied as a potential target for pain treatment (25). *FMR1* knockout mice that produce no FMRP show decreased neuropathic pain, protection from nociceptive sensitization (26–28) or IL-6 induced allodynia (29), and protection from pain-induced emotional sequelae such as depression (24). FMRP is hypothesized to mediate translational control over allodynia and persistent nociceptive sensitization (29). The primary pathophysiology of FXTAS is thought to be through increased mRNA production and toxicity. Despite increased mRNA production, FMRP is paradoxically decreased as CGG repeats increase, likely secondary to less efficient translation due to excessive repeats (30, 31). Because individuals with FXTAS frequently complain of chronic pain we have surveyed 104 patients with FXTAS to clarify what types of pain occur in this disorder.

## Materials and Methods

### Participants

This study was conducted at the Fragile X Research and Treatment Center of the MIND Institute, University of California, Davis Medical Center, with participants in the Genotype-Phenotype Relationships in Fragile-X families data set. Participants with FXTAS were identified through families of known members affected by fragile X syndrome, referral from physicians, and *via* self-referral. All participants gave written informed consent from the UCD IRB and were categorized by the stages of FXTAS (32): stage (0) no tremor/ataxia; (1) questionable tremor and/or ataxia; (2) mild tremor and/or balance problems with minimal interference in activities of daily living (ADLs); (3) moderate tremor and/or ataxia with significant interference in ADLs; (4) severe tremor and/or ataxia requiring a cane or walker; (5) requiring daily use of wheelchair; (6) bedridden. We have eight participants who do not meet the diagnostic criteria for FXTAS (2), although all have premutation and subtle neurological symptoms apart from one patient who has the MCP sign without tremor or ataxia, a phenomenon previously reported in five males (33). These patients likely represent the most subtle end of the spectrum involving neurological deficits in the premutation and are included for comparison purposes. Participants who reported medical conditions such as a history of stroke, head injury, or primary language other than English were excluded from the study.

### Clinical Assessment and Molecular Analysis

Participants underwent medical and neurological evaluation to assess FXTAS diagnosis, stage, and *FMR1* molecular studies for CGG repeat size and mRNA level. A medical history was taken at the time of visit to document FXTAS phenotype and symptoms including tremor and ataxia in addition to age of onset and severity, current and past medication use, self-reported anxiety and depression, musculoskeletal and autoimmune problems such as osteopenia, osteoporosis, osteoarthritis, rheumatoid arthritis, fibromyalgia, lupus, ANA positivity, and other immunological diseases. We collected data on restless legs syndrome, severe cramps, disc/spine problems, and muscle pain. Migraine with and without aura, age of onset, and frequency was collected along with symptoms of neuropathy, allodynia, back pain, and chronic pain in general. Activation ratio was measured using ratios of signal intensity with Southern blot as previously described (34). *FMR1* CGG repeat allele length was quantified using a combination of both PCR and Southern blot analysis as previously described (35).

### Medication Grouping

Pain medication categories were separated into non-overlapping categories of opiate analgesics, non-opiate analgesics, cannabinoids, anesthetics, antimigraine, nerve pain, and muscle pain/relaxation. Non-opiate analgesics included non-steroidal anti-inflammatory drugs (NSAIDs) and acetaminophen. Anesthetics included lidocaine. The category of antimigraine medications did not overlap with other medications categories and contained triptans, topiramate, and botulinum injections. Nerve pain medications were based on first line treatments and include tricyclics, serotonin and norepinephrine reuptake inhibitors (SNRIs), and anti-epileptic drugs (AEDs) including gabapentin, pregabalin, and carbamazepine. Muscle pain/relaxation medications included antispasticity and antispasmodic agents such as baclofen, tizanidine, and cyclobenzaprine.

### Statistical Analysis

Statistical analyses of data were performed with an open-source R software. Results were expressed as mean ± standard deviation (or error) of mean for continuous variables and proportion (%) for categorical variables. For quantitative variables, group differences in means or medians were determined by *t*-test, analysis of variance (ANOVA), or Kruskal-Wallis test as appropriate. For categorical variables, proportions were compared between groups using *t*-test for proportions, chi-squared test, or Fisher's exact test as appropriate. Linear regression analysis was performed to assess associations between two variables with the main effects of the predictor and gender and their interaction term included in the linear models. Missing values were excluded from the statistical inference tests. Two-tailed *p* < 0.05 were considered statistically significant as appropriate.

## Results

### Clinical Characteristics

The participants are 104 individuals (41 female and 63 male) with FXTAS and the premutation confirmed *via* molecular studies. No significant gender difference was found for race and ethnicity (*p* = 0.513), education level (*p* = 0.199), age (*p* = 0.743), CGG repeat size (*p* = 0.266), or FXTAS stage (*p* = 0.404). The mean (SD) age of females was 68.1 (8.8) years and age of males was 67.5 (8.0) years. Race and ethnicity are primarily white (100% female vs. 95.7% male). Of the 41 females in the study, 34 (85%) are FXTAS stage >2. Of the 63 males, 57 (90.5%) are FXTAS stage >2. The mean CGG repeat size for females is 87 (SD = 18), and the mean for males is 90 (SD = 16). Refer to Table 1. There is a significant difference in FXTAS diagnostic categories (*p* = 0.001) with more males diagnosed with “Definite FXTAS” than females (48.3% male vs. 9.8% female). Females are more represented in the Probable category (30.0% male vs. 58.5% female).

**Table 1:** Summary statistics [Mean ± SD or N(%)] of patient characteristics and age onset (years) of symptoms.

	**Total (*N* = 104)**	**Female (*N* = 41)**	**Male (*N* = 63)**	***p-value* (gender difference)**
Age of visit (years)	67.8 ± 8.3	68.1 ± 8.8	67.5 ± 8.0	0.7431
**FXTAS stage**
0/1	12 (11.7%)	6 (15.0%)	6 (9.5%)	0.5305
2	23 (22.3%)	10 (25.0%)	13 (20.6%)	0.6335
3	49 (47.6%)	17 (42.5%)	32 (50.8%)	0.4269
4	13 (12.6%)	4 (10.0%)	9 (14.3%)	0.7619
5	6 (5.8%)	3 (7.5%)	3 (4.8%)	0.6751
CGG repeat size	89 ± 16.4	86.7 ± 17.7	90.4 ± 15.5	0.2802
**Age of onset (years)**
Tremor	58.61 ± 9.16	58.83 ± 9.36	58.44 ± 9.13	0.8591
Ataxia	60.71 ± 9.44	59.81 ± 11.82	61.33 ± 7.48	0.5011
Chronic pain	51.36 ± 17.3	52.27 ± 19.27	50.15 ± 15	0.7541
Peripheral neuropathy symptoms	57.36 ± 11.36	55.62 ± 12.92	59 ± 9.79	0.4021
Migraines	28.73 ± 17.63	32.62 ± 19.22	21.8 ± 12.49	0.1441

### Age of Symptom Onset

Mean age of onset of migraine is 28.7 years (SD = 17.6), chronic pain is 51.4 years (SD = 17.3), peripheral neuropathy is 57.4 years (SD = 11.4), tremor is 58.6 years (SD = 9.2), and ataxia is 60.7 (9.4), and no gender differences were found (see Table 1).

**Figure 1:**
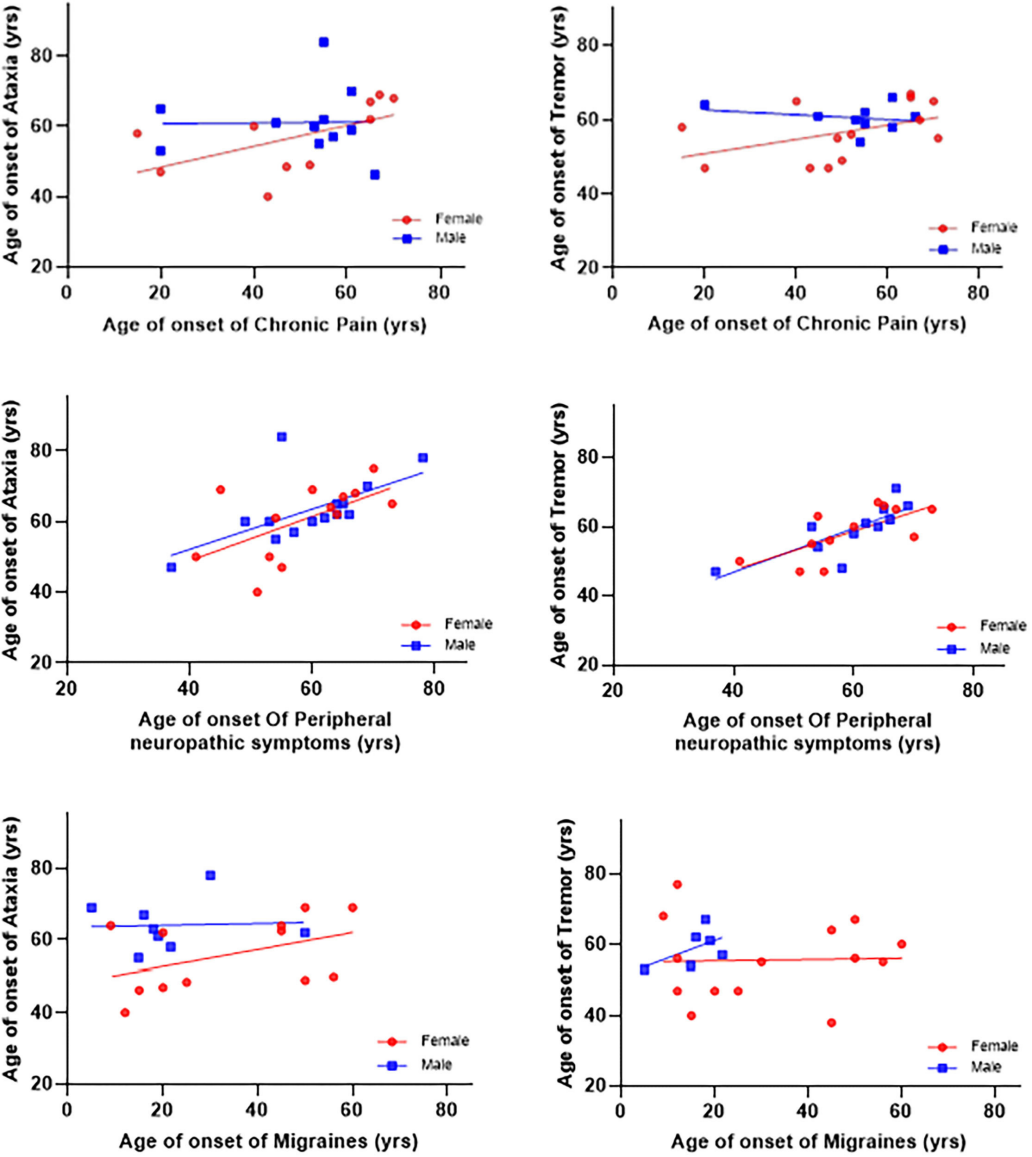
Associations between ages of onset of symptoms and ataxia and tremor. The age of onset of peripheral *neuropathy* symptoms was positively associated with age of onset ataxia (β = 0.56 ± 0.17, *p* = 0.004) and tremor (β = 0.56 ± 0.17, *p* = 0.004). There were no significant associations between the onset of chronic pain and migraines with the onset of ataxia and tremor.

The age of onset of peripheral *neuropathy* symptoms was positively associated with the age of onset of ataxia (β = 0.56 ± 0.17, *p* = 0.004) and tremor (β = 0.56 ± 0.17, *p* = 0.004). There were no significant associations between the onset of chronic pain and migraine with the onset of ataxia and tremor (see Table 2 and Figure 1). In addition, there were no gender differences for any of those associations.

**Table 2:** Associations between age onset of symptoms and age onset of ataxia and tremor.

**Symptom**	**Ataxia**				**Tremor**			
	**Main effect**		**Gender difference**		**Main effect**		**Gender difference**	
	**β** **(SE)**	***p-value***	**β** **(SE)**	***p-value***	**β** **(SE)**	***p-value***	**β** **(SE)**	***p-value***
Chronic pain	0.3 (0.17)	0.091	−0.29 (0.26)	0.280	0.19 (0.10)	0.061	−0.26 (0.18)	0.178
Peripheral neuropathy symptoms	0.63 (0.25)	**0.019**	−0.06 (0.34)	0.868	0.56 (0.17)	**0.004**	0.08 (0.25)	0.761
Migraines	0.24 (0.14)	0.120	−0.21 (0.29)	0.480	0.02 (0.15)	0.908	0.48 (0.81)	0.567

*Bold values are p-values that have reached significance (P < 0.05) and are meant to draw the reader to the most important values*.

### Pain Prevalence in FXTAS

There were significant gender differences in the prevalence of several symptoms. Back pain was significantly higher in females than males (48.5 vs. 23.4%, *p* = 0.008), as was migraine (43.9 vs. 14.5%, *p* = 0.0008), fibromyalgia (26.8 vs. 0%, *p* = 0.0071), thyroid problems (34.2 vs. 14.5%, *p* = 0.0182), osteoarthritis (65.9 vs. 43.5%, *p* = 0.027), allodynia (20 vs. 2.0%, *p* = 0.008), peripheral neuropathy pain (43.9 vs. 25.4%, *p* = 0.0488), and anxiety (82.9 vs. 39.7%, *p* < 0.001). No significant gender differences were found in autoimmune problems, musculoskeletal pain, or depression (see Table 3).

**Table 3:** Prevalence [N of Yes (%)] of symptoms (Yes/No).

**Symptoms**	**Total (*N* = 104)**	**Female (*N* = 41)**	**Male (*N* = 63)**	***p-value* (gender difference)**
Back pain	27 (33.8%)	16 (48.5%)	11 (23.4%)	**0.008**
Migraines	27 (26.2%)	18 (43.9%)	9 (14.5%)	**0.0008**
Fibromyalgia	11 (10.9%)	11 (26.8%)	0 (0%)	**0.0071**
Autoimmune	10 (12.3%)	6 (16.7%)	4 (8.9%)	0.2301
Thyroid problems	22 (22%)	13 (34.2%)	9 (14.5%)	**0.0182**
Osteoarthritis	54 (52.4%)	27 (65.9%)	27 (43.5%)	**0.027**
Musculoskeletal pain	86 (88.7%)	37 (92.5%)	49 (86%)	0.3077
Peripheral neuropathy pain	33 (33.0%)	18 (43.9%)	15 (25.4%)	**0.0488**
Allodynia	8 (9.5%)	7 (20.0%)	1 (2.0%)	**0.008**
Anxiety	59 (56.7%)	34 (82.9%)	25 (39.7%)	**<0.001**
Depression	26 (25.2%)	10 (24.4%)	16 (25.8%)	0.413

*Bold values are p-values that have reached significance (P < 0.05) and are meant to draw the reader to the most important values*.

### CGG Repeat and Pain

Among participants who experienced pain, there were no gender differences in CGG repeat length. Among participants who had experienced autoimmune problems, there was a significant gender difference in CGG repeat length. Females with autoimmune had significantly lower CGG repeats than that of males (76.5 ± 12.9 female vs. 105.5 ± 26.5 male, *p* = 0.032). Refer to Supplementary Table 4.

### Musculoskeletal vs. Neuropathic Pain

Overall, the prevalence of patients with musculoskeletal pain (severe cramps + muscle pain + arthritis + back pain + disc or spine problems) was significantly higher than the prevalence of patients with peripheral neuropathic pain (PNP) (88.4 vs. 33.7%, *p* < 0.0001). The difference between the proportion with neuropathic pain and the proportion with musculoskeletal pain was significant in both males and females, *p* = <0.0001 (85.5 vs. 25.5%) and *p* = <0.0001 (92.5 vs. 45%), respectively (see Supplementary Table 5).

### Activation Ratio and Pain

The data does not provide evidence that activation ratio is significantly associated with either neuropathic pain or musculoskeletal pain, *p* = 0.241 and *p* = 0.770, respectively. The mean activation ratio for those with neuropathic pain is 0.52, while the mean activation ratio is 0.60 for those without neuropathic pain. The mean activation ratio for those with musculoskeletal pain is 0.57 while the mean activation ratio is 0.61 for those without musculoskeletal pain (see Supplementary Table 6).

### Prevalence of Pain Medicine Usage

Females with FXTAS are significantly more likely to be taking any pain medication (58.5% females vs. 36.5% males, *p* = 0.0271) as well as nerve pain medication (31.7% females vs. 14.3% males, *p* = 0.331). No significant gender differences were found for other medication types. Refer to Supplementary Table 7.

## Discussion

### Findings

Both men and women with FXTAS experience chronic neuropathic and musculoskeletal pain. However, women with FXTAS experience significantly more chronic pain than men and take more pain medications. It's currently unclear why women with FXTAS experience more chronic pain than men, particularly central sensitivity syndromes, neuropathic pain, and back pain. Possible mechanisms include differences in endocrinology, autoimmune conditions, connective tissue, inflammation, and neuropsychiatric differences.

### Central Sensitivity

Females with FXTAS were more likely to have conditions that fit under the umbrella of central sensitivity syndromes, characterized by altered pain regulation such as fibromyalgia, allodynia, chronic pain, and chronic headaches (6). We also found that the defining symptom of central sensitization, allodynia, is significantly higher in women with FXTAS (*p* = 0.008). Sensitization occurs following intense or chronic peripheral noxious stimuli, tissue injury, or nerve damage that keeps the peripheral nervous system in a constant state of activity. Chronic pain conditions commonly known to lead to hypersensitivity such as back pain, arthritis, and fibromyalgia are significantly prevalent among women with FXTAS. Central sensitivity symptoms are heavily influenced by psychological stressors (21), which are significantly worse for females with FXTAS in our cohort. Despite the known role of FMRP in altering sensitization of pain and neuropathy in animal models, we did not find that these pain phenotypes correlated with CGG repeat length.

### Migraine

We found a quarter of participants (26.2%) reported experiencing migraines, with rates significantly higher among females than males (43.9% females vs. 14.5% males, *p* = 0.0008). Age of migraine onset had no bearing over age of ataxia and tremor onset. This confirms a previous study on *FMR1* premutation carriers and the prevalence of migraines (13). Author Au hypothesized that migraines in FXTAS are related to mitochondrial dysfunction leading to increased oxidative stress, which worsens with age. Several chronic pain syndromes prevalent in female *FMR1* premutation carriers are comorbid with migraine including fibromyalgia (36), allodynia (37), and chronic fatigue syndrome (36), suggesting diffuse alterations to the nociceptive nervous system. Individuals with migraines are also at an increased risk for anxiety and depression (38).

### Psychiatric

We found that women with FXTAS self-reported significantly more anxiety symptoms (82.9% females vs. 39.7% males, *p* = 0.001); depressive symptoms were present in a quarter of participants (24.4% female vs. 25.8% male). This finding is consistent with previous studies that found women with FXTAS have more depression and emotional symptoms (4, 7). The relationship between pain and psychopathology is multi-directional; chronic pain is a strong predictor of both onset and persistence of comorbid psychiatric disorders in general, and vice versa, psychiatric disorders are also a powerful predictor of chronic pain persistence (39–41). Emotional states can also amplify the pain felt from central sensitivity syndromes (21). Women with FXTAS may report more chronic pain partly because that pain is viewed through a lens intensified by symptoms of anxiety, which play a role in pain perception (42). How significant a role mental health plays in FXTAS across gender may also be influenced by reporting bias, as men are less likely to disclose mental health problems than women in general (43).

### Hormonal

Consistent with previous research on bone density in premutation carriers (44, 45), females were found to have more skeletal related problems such as osteopenia, osteoporosis, and osteoarthritis. With respect to pain, women with FXTAS reported significantly more back pain than men (48.5% of women vs. 23.4% of men, *p* = 0.008). Refer to Table 3. Post-menopausal women in the general population do tend to have higher incidence of back pain than men (46, 47), but percentages are similar between men and women in the 65–74 age group within the US (48). The cause of these gender differences is likely secondary to hormonal differences in post-menopausal women. The role estrogen plays in bone density is additionally important for women with the premutation because the mean age of natural menopause is reduced by about 5 years, from the typical age of about 51 to 46. Separate from this overall earlier menopause, 20% of carriers (49) can develop fragile X-associated primary ovarian insufficiency (FXPOI) with typically onset at 33 years (50). This directly decreases the lifetime exposure of estrogen and decreases bone mineral density, as early menopause is associated with osteoporosis (51). The significantly increased back pain among women is likely secondary to decreased bone density-related sequelae, which can have a devastating impact on women with FXTAS.

### Neuropathy

More women report feeling peripheral neuropathic pain than men (43.9 vs. 25.4%, *p* = 0.0488). Women were also more likely to be taking medications that treat nerve pain (31.7 vs. 14.3%, *p* = 0.0331), however some of these medications overlap with treatment for anxiety, which was also significant among women with FXTAS. We did not find CGG repeat number correlated with nerve pain in men or women. Nerve conduction studies have shown men and women have similar sensory nerve fiber abnormalities (52), but men have additional motor nerve abnormalities in conduction velocity and latency (53). Pathogenesis of neuropathy is related to RNA toxicity causing the creation of intracellular inclusion bodies in neurons and glial cells (54) and peripheral tissues (55, 56) that causes axonal dysfunction.

Consistent with previous research that neuropathy is a presenting feature of FXTAS (8), we found the age onset of peripheral neuropathy was positively associated with the age onset of ataxia (β = 0.63 ± 0.25, *p* = 0.019) and tremor (β = 0.56 ± 0.17, *p* = 0.004), with no gender difference. This further supports the recommendation to consider a FXTAS diagnosis in a patient presenting with neuropathy and a family history of intellectual disability, premature ovarian failure, autism, or movement disorder (8).

### Connective Tissue

We found that musculoskeletal pain is significantly more prevalent than peripheral neuropathy pain (88.4 vs. 33.7%, *p* < 0.0001) in FXTAS. There was no significant gender difference in musculoskeletal pain (92.5 vs. 85.5%, *p* = 0.3077). Of interest, every patient that reported neuropathic pain also reported musculoskeletal pain, meaning all who experienced neuropathic pain also reported arthritis, back pain, severe cramping, or muscle pains. However, 54.7% of those with musculoskeletal pain did not have neuropathic pain (see Supplementary Table 6). Clinically, premutation carriers can manifest connective tissue-related features such as hyperextensible finger joints, large ears, and connective tissue dysplasia that are more subtle than those seen in FXS (57). Case report evidence including five females with premutation and Ehlers Danlos syndrome phenotype suggests there may be related commonalities in pathogenesis (58). Possible pathophysiologic mechanisms hypothesized include FMRP deficiency, mRNA toxicity, and sex effects (58). FMRP is known to regulate multiple connective tissue pathways including elastin, actin, and matrix metalloproteinase (MMP9), a class of enzymes involved in bone development, wound healing, and pathology such as arthritis and intracerebral hemorrhage (59, 60). Levels of connective tissue involvement correlate with level of FMRP depletion in FXS and premutation CGG repeats > 120 are more likely to be associated with connective tissue problems (61). Because the FXTAS population has minimal alterations to FMRP which result in altered regulation of these pathways, this mechanism could contribute to generating chronic musculoskeletal pain in FXTAS.

### Autoimmune

We did not find a significant gender difference (*p* = 0.2301) in rheumatoid arthritis, systemic lupus erythematosus, or antinuclear antibodies positivity, but the prevalence for these conditions was quite low. Confirming previous research, we found FXTAS women are more likely to have fibromyalgia (26.8 vs. 0%, *p* = 0.0071) and thyroid problems (34.2 vs. 14.5%, *p* = 0.0182). Fibromyalgia is generally thought to be immune mediated (62), but its etiology remains unclear with some evidence linking it to small nerve fiber neuropathy (63) and central sensitization (64). Thyroid problems consisted almost entirely of autoimmune causes, including hypo and hyperthyroid states. Females with autoimmune conditions had significantly lower CGG repeats than that of males, likely because women are more likely to have autoimmune conditions at baseline. Autoimmune conditions and inflammation have strong links to pain and the primary pathophysiology of FXTAS involves RNA toxicity and inflammation. Inflammatory mediators from damaged cells induce pain and sensitization. Local tissue inflammation can result in hypersensitivity to pain *via* secondary hyperalgesia, where inflammatory mediators diffuse to uninjured nearby tissues. Females produce a larger proinflammatory immune response to tissue damage than males (65). Females with FXTAS may have increased chronic pain because they develop more inflammation contributing to sensitization and maintenance of pain.

### Pain Treatment

Non-opiate pharmacologic therapies can be selected based on type of pain (nociceptive, neuropathic, central sensitization, and combination) or by targeting comorbid conditions.

### Psychopharmacologic Overlap

Considering both psychiatric and pain symptoms of a patient is crucial as psychiatric disorders can exacerbate pain conditions and impede treatment adherence (66). Treatment of depression and anxiety in fragile X-associated neuropsychiatric disorders (FXAND) typically involves SSRIs or SNRIs (15, 67). In addition to targeting symptoms of depression and anxiety, antidepressants such as tricyclics (TCAs), SSRIs, SNRIs, and norepinephrine dopamine reuptake inhibitors (NDRIs) have evidence for use in chronic pain and are commonly used in the treatment of patients with FXTAS. Of the antidepressants, evidence suggests SNRIs and TCAs are the most effective for treating chronic neuropathic pain (68) as well as centralized pain (21). There is also strong evidence for the use of anticonvulsants pregabalin and gabapentin, and with moderate evidence for use of SSRIs (21).

We recommend avoidance of opiates to treat chronic pain associated with FXTAS. Opiates are commonly used to treat pain in the general population and FXTAS population, but anecdotal evidence suggests those on opioids can have a faster progression of their FXTAS symptoms (69). Opiates are reported to trigger white matter changes in chronic users (70, 71). *In vitro* evidence suggests premutation neurons are more vulnerable to environmental toxins than normal neurons (67). Drug and alcohol use has been reported to be increased in premutation carriers compared to controls (72, 73), which has resulted in opiate overdose (74). Long-term opiate use leads to tolerance, dependence, withdrawal symptoms, and can worsen chronic pain through development of opiate-induced hyperalgesia (75).

Topical treatments can be recommended for chronic pain such as diclofenac and ketoprofen for musculoskeletal issues (76). For osteoarthritis, topical NSAIDs, low-concentration capsaicin, or topical rubefacients. For neuropathic pain, topical lidocaine or high-concentration capsaicin can be used.

### Emerging Pharmacotherapy

CBD is a compound typically derived from hemp that targets CB1, CB2 receptors and is an allosteric modulator of μ- and δ-opioid receptors. Evidence in *FMR1* knockout mice suggests CB2 receptor is necessary for protection against neuropathic pain, raising interest in targeting this receptor for treatment (77). A controlled trial examined pain in patients with multiple sclerosis, spinal cord, and other neurological conditions found pain control improved with CBD (78) in addition to two other studies that found pain relief (79, 80). CBD is also being explored as an intervention in FXS (81) in an open-label trial (82) because of its benefits on sleep quality, anxiety (83), and cognitive impairment (84).

Curcumin is a polyphenol with anti-inflammatory properties extracted from turmeric that has shown promising preclinical results in treating FXTAS (85) but has not been studied clinically. Curcumin has a notable anti-inflammatory effect and pain relief for the treatment of general pain syndrome and osteoarthritis (86). However, more comprehensive long-term studies are needed, as many of the controlled trials are of low quality with industry funding. Sulforaphane is another dietary supplement with antioxidant/anti-inflammatory properties that was found to improve markers of oxidative stress and mitochondrial function in fibroblasts from FXTAS patients (87), but has not been studied for pain symptoms in FXTAS.

Allopregnanolone, a natural neurosteroid and positive allosteric modulator at GABA-A, has been studied as a safe therapy for neuropathic pain (88, 89), treatment for post-partum depression (90), and has been studied in an open-label trial for treatment of FXTAS in six patients with limited benefits on executive function and neuropathy symptoms (91). Allopregnanolone is a promising candidate for FXTAS because it improves mitochondrial dysfunction and acts to prevent reactive oxidative species (ROS) overproduction, a primary pathological mechanism driving FXTAS phenotype. ROS produced in mitochondria have been shown to contribute to central sensitization associated with pain (92). Allopregnanolone was able to protect neuronal cells against oxidative stress through improved mitochondrial antioxidant activity (93).

### Non-pharmacologic

For a non-pharmacologic approach, counseling and therapy for chronic pain and psychiatric conditions has been helpful. The cognitive behavioral therapy approach to pain teaches patients skills to anticipate pain and divert attention to other thoughts, helping the patient better cope with pain (94). We also recommend daily exercise to stimulate neurogenesis, improve mitochondrial function, and decrease chronic pain (67). Focusing on maintaining sleep quality can have an impact on pain (95). We recommend other techniques for reducing overall stress such as biofeedback for relaxation, which can lead to improvements in various pain-related outcomes (96). Mindfulness stress reduction has evidence for reducing chronic pain and improving quality of life (97). Acupuncture has been studied in hundreds of controlled trials and appears to be effective for chronic pain, particularly back pain (98) and headaches (99).

### Study Limitations

The limitations of this study include a sample bias, as the cohort consisted of participants with higher socioeconomic status and majority with white ethnicity. Data on anxiety and depression are self-reported and the symptomatology of pain is recorded through medical interviews. Future studies characterizing pain would benefit from measures of intensity. Furthermore, participants were enrolled through referral from physicians or family members and thus this cohort might not be a true representative of individuals with FXTAS, but the more affected ones. In addition, men are less likely to disclose health issues, and at more advanced FXTAS stages may not be able to assess themselves accurately.

## Conclusion

In conclusion, our results have important clinical implications for the treatment of female premutation carriers with FXTAS who have increased back pain, neuropathy, and central sensitization related pain. Pain is a significant finding in FXTAS and should be questioned in the medical history for all premutation carriers and treated appropriately.

## Data Availability Statement

The datasets presented in this study can be found in online repositories. The names of the repository/repositories and accession number(s) can be found at: https://redcap.ucdmc.ucdavis.edu/redcap/, CTSC#3704. Further enquiries can be directed to the corresponding author.

## Ethics Statement

The studies involving human participants were reviewed and approved by Institutional Review Board at the University of California Davis. The patients/participants provided their written informed consent to participate in this study.

## Author Contributions

ES, DJ, and RH conceived of the presented idea and questions. DJ developed the idea and wrote the manuscript with support from ES, RH, KK, and MP. KK and MP performed statistical computations and created figures and tables. YM, RH, ES, AS, and DJ collected data. FT conducted molecular studies. All authors contributed to the article and approved the submitted version.

## Funding

This work was supported by NICHD Grant Number: HD036071, the Tides foundation and the MIND Institute IDDRC (Grant Number: U54 HD079125).

## Conflict of Interest

RH has received funding from Zynerba and the Azrieli foundation for carrying out treatment studies in patients with fragile X syndrome. FT has received funding from Zynerba and Asuragen, Inc. The remaining authors declare that the research was conducted in the absence of any commercial or financial relationships that could be construed as a potential conflict of interest.

## Publisher's Note

All claims expressed in this article are solely those of the authors and do not necessarily represent those of their affiliated organizations, or those of the publisher, the editors and the reviewers. Any product that may be evaluated in this article, or claim that may be made by its manufacturer, is not guaranteed or endorsed by the publisher.
