# Supplementary material for: Increased Pain Symptomatology Among Females vs. Males With Fragile X-Associated Tremor/Ataxia Syndrome
Source: Front Psychiatry. 2022 Jan 20;12:762915. doi: 10.3389/fpsyt.2021.762915 (PMC8811376; doi:10.3389/fpsyt.2021.762915)
Supplement: Supplementary file 1 [file Table_1.DOCX]

Supplementary Material

# Supplementary Tables

| Table 4*. CGG repeat size (Mean ± SD) by pain conditions (Painful: Yes/No) and gender | | | | | | | | |
| --- | --- | --- | --- | --- | --- | --- | --- | --- |
| Pain condition | Pain, Yes | | | | Pain, No | | | |
|  | Total | Female | Male | *p-value (gender difference)* | Total | Female | Male | *p-value (gender difference)* |
| Back Pain | 90.4 ± 22.9 | 88.8 ± 22.1 | 92.7 ± 24.9 | 0.693 | 88.3 ± 11.4 | 87.4 ± 12.8 | 88.8 ± 10.9 | 0.675 |
| Migraines | 88.1 ± 16.3 | 90.2 ± 17.6 | 84 ± 13.2 | 0.607 | 89.3 ± 16.7 | 84 ± 17.6 | 91.6 ± 15.9 | 0.140 |
| Fibromyalgia | - | 89.9 ± 24.1 | na (n=0) | na | 89.2 ± 15.6 | 85.6 ± 15 | 91 ± 15.7 | 0.183 |
| Autoimmune | 88.1 ± 23.5 | 76.5 ± 12.9 | 105.5 ± 26.5 | 0.032 | 88.3 ± 15 | 90 ± 17.4 | 87 ± 13 | 0.389 |
| Musculoskeletal Pain | 89.1 ± 17.1 | 87.9 ± 18.2 | 90.1 ± 16.3 | 0.682 | 86.5 ± 13.9 | 74.7 ± 4.9 | 91 ± 13.6 | 0.102 |
| Peripheral Neuropathy Pain | 87.7 ± 16.6 | 87.8 ± 17 | 87.5 ± 16.7 | 0.986 | 89.5 ± 16.5 | 85.9 ± 18.5 | 91.4 ± 15.2 | 0.111 |
| *Due to small Ns, Kruskal-Wallis tests were performed. | | | | | | | | |

Page Break

| Table 5.  Cross prevalence* (N of Yes (%)) of Musculoskeletal (MSK) pain and Neuropathic (PNP) pain (Painful: Yes/No) by gender | | | | |
| --- | --- | --- | --- | --- |
| Condition | Total (N=95) | Female (N=40) | Male (N=55) | p-value (gender difference) |
| Musculoskeletal Pain | 84 (88.4%) | 37 (92.5%) | 47 (85.5%) | 0.2937 |
| Peripheral Neuropathy Pain | 32 (33.7%) | 18 (45.0%) | 14 (25.5%) | 0.0466 |
| Both MSK and PNP | 32 (33.7%) | 18 (45.0%) | 14 (25.5%) | 0.0466 |
| MSK Only | 52 (54.7%) | 19 (47.5%) | 33 (60.0%) | 0.2262 |
| PNP Only | 0 (0%) | 0 (0%) | 0 (0%) | na |
| Neither | 11 (11.6%) | 3 (7.5%) | 8 (14.5%) | 0.2937 |
| Female (N=40) | | Musculoskeletal Pain | | |
|  |  | Pain, Yes | Pain, No | Total |
| Peripheral Neuropathy Pain | Pain, Yes | 18 | 0 | **18 (45%)** |
|  | Pain, No | 19 | 3 | 22 (55%) |
|  | Total | **37 (92.5%)** | 3 (7.5%) | 40 |
| Male (N=55) | | Musculoskeletal Pain | | |
|  |  | Pain, Yes | Pain, No | Total |
| Peripheral Neuropathy Pain | Pain, Yes | 14 | 0 | **14 (25.5%)** |
|  | Pain, No | 33 | 8 | 41 (74.5%) |
|  | Total | **47 (85.5%)** | 8 (14.5%) | 55 |
| *This analysis is limited to participants (N=95) who answered both questions. | | | |  |

Page Break

| Table 6. Comparison of activation ratio by pain (Yes/No) in females | | | | | | |  | |  |
| --- | --- | --- | --- | --- | --- | --- | --- | --- | --- |
|  | Total | | Yes | | No | | *p-value*(Yes vs. No) | |  |
| Peripheral Neuropathy Pain | 0.57 ± 0.22 | | 0.52 ± 0.25 | | 0.60 ± 0.19 | | 0.241 | |  |
| Musculoskeletal Pain | 0.57 ± 0.22 | | 0.57 ± 0.22 | | 0.61 ± 0.25 | | 0.77 | |  |
|  |  | |  | |  | |  | |  |
| Table 7. Proportion (N (%) of Yes) of participants who took medication in a given category, by gender | | | | | | | | | |
|  | | Total (N=104) | | Female (N=41) | | Male (N=63) | | *p-value*(gender difference) | |
| Opiate Analgesics | | 10 (9.6%) | | 5 (12.2%) | | 5 (7.9%) | | 0.4715 | |
| Non-opiate Analgesics | | 21 (20.2%) | | 7 (17.1%) | | 14 (22.2%) | | 0.5221 | |
| Cannabinoids | | 4 (3.9%) | | 3 (7.3%) | | 1 (1.6%) | | 0.1388 | |
| Anesthetics | | 1 (1.0%) | | 1 (2.4%) | | 0 (0%) | | 0.2113 | |
| Antimigraine | | 5 (4.8%) | | 2 (4.9%) | | 3 (4.8%) | | 0.976 | |
| Nerve Pain | | 22 (21.2%) | | 13 (31.7%) | | 9 (14.3%) | | 0.0331 | |
| Muscle/Relaxation/Back Pain | | 2 (1.9%) | | 2 (4.9%) | | 0 (0%) | | 0.0767 | |
| Any Pain Medication | | 47 (45.2%) | | 24 (58.5%) | | 23 (36.5%) | | 0.0271 | |
